# Effects of buoyancy on the dispersion of drugs released intrathecally in the spinal canal

**DOI:** 10.1017/jfm.2024.297

**Published:** 2024-04-19

**Authors:** J. Alaminos-Quesada, C. Gutiérrez-Montes, W. Coenen, A.L. Sánchez

**Affiliations:** 1 Department of Mechanical and Aerospace Engineering, University of California San Diego, La Jolla, CA, 92093-0411, USA; 2 Department of Mechanical and Mining Engineering, University of Jaén, Jaén, 23071, Spain; 3 Andalusian Institute for Earth System Research, University of Jaén, Campus de las Lagunillas, Jaén, 23071, Spain; 4 Grupo de Mecánica de Fluidos, Departamento de Ingeniería Térmica y de Fluidos, Universidad Carlos III de Madrid, Leganés, 28911, Spain

**Keywords:** biomedical flows

## Abstract

This paper investigates the transport of drugs delivered by direct injection into the cerebrospinal fluid (CSF) that fills the intrathecal space surrounding the spinal cord. Because of the small drug diffusivity, the dispersion of neutrally buoyant drugs has been shown in previous work to rely mainly on the mean Lagrangian flow associated with the CSF oscillatory motion. Attention is given here to effects of buoyancy, arising when the drug density differs from the CSF density. For the typical density differences found in applications, the associated Richardson number is shown to be of order unity, so that the Lagrangian drift includes a buoyancy-induced component that depends on the spatial distribution of the drug, resulting in a slowly evolving cycle-averaged flow problem that can be analysed with two-time scale methods. The asymptotic analysis leads to a nonlinear integro-differential equation for the spatiotemporal solute evolution that describes accurately drug dispersion at a fraction of the cost involved in direct numerical simulations of the oscillatory flow. The model equation is used to predict drug dispersion of positively and negatively buoyant drugs in an anatomically correct spinal canal, with separate attention given to drug delivery via bolus injection and constant infusion.

## 1. Introduction

**Figure 1.**
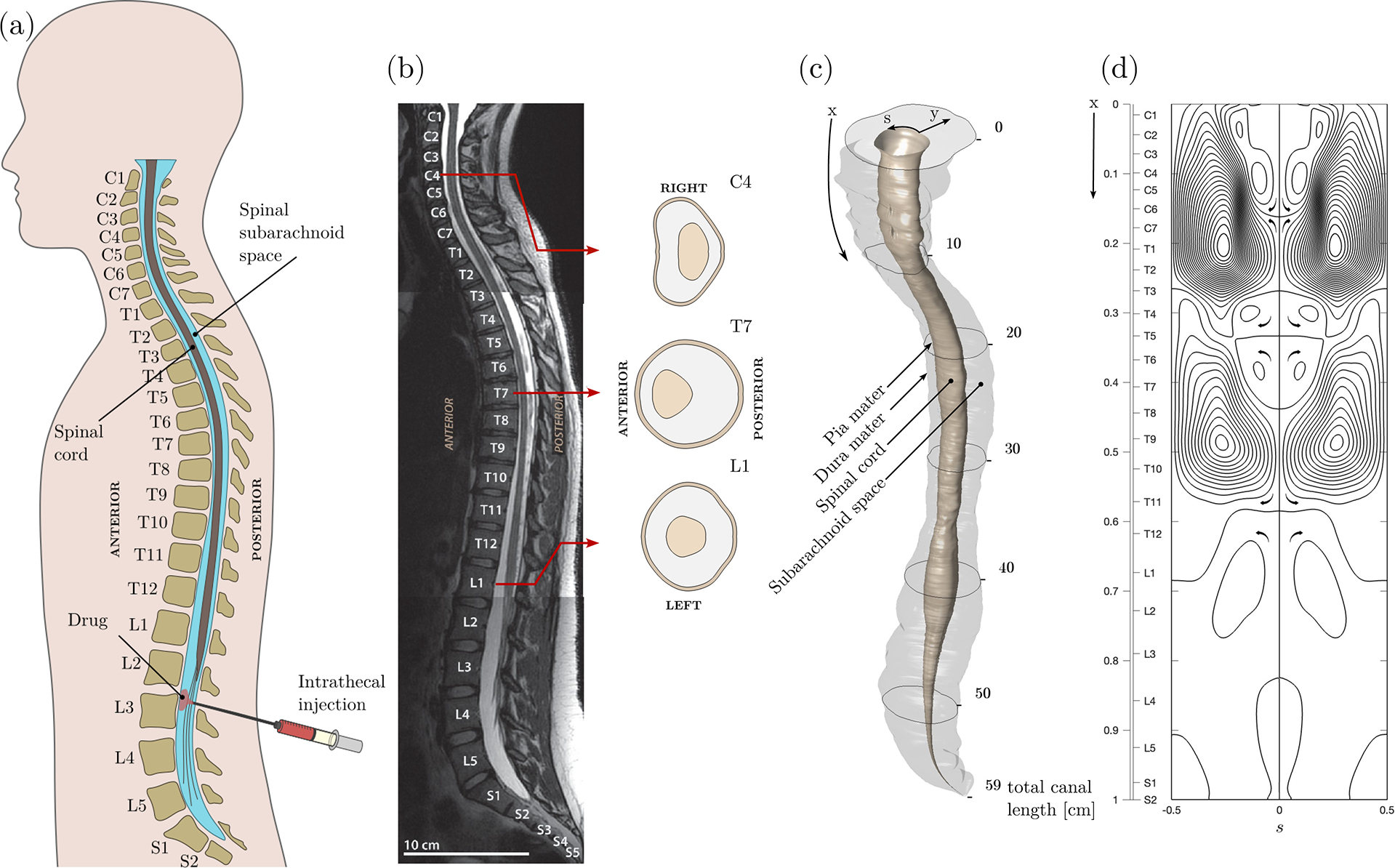
The spinal canal. (*a*) A schematic showing the typical intrathecal injection location. (*b*) Sagittal T2-weighted magnetic resonance (MR) image of the spine in a subject in the supine position, including cross-sectional views at three different locations. (*c*) Transversely stretched three-dimensional view of the spinal canal obtained after Gaussian smoothing the MR images, with an indication of the bounding surfaces and the dimensionless coordinate system used in the model derivation. (*d*) Streamlines of the Lagrangian flow projected onto the dimensionless plane *x*–*s* (see § 6).

The subarachnoid space (SAS) surrounding the spinal cord is filled with cerebrospinal fluid (CSF), a colourless Newtonian fluid whose density ρ and kinematic viscosity v are very similar to those of water. The CSF moves in response to the cyclic pressure variations induced by the blood pulsations in the cranial cavity and to the abdominal pressure variations associated with the respiratory cycle (Linninger et al. 2016; Kelley & Thomas 2023). CSF motion plays a fundamental role in the physiological function of CSF as a vehicle for the transport of hormones, nutrients and neuroendocrine substances (Greitz, Franck & Nordell 1993; Greitz & Hannerz 1996; Pollay 2010), and also facilitates the dispersion of drugs delivered by direct injection into the SAS (Hettiarachchi et al. 2011). This medical procedure, known as intrathecal drug delivery (ITDD), has been used since the early 1980s to bypass the blood–brain barrier, facilitating the administration of analgesics, chemotherapy and enzymes to the central nervous system (Onofrio, Yaksh & Arnold 1981; Greene 1985; Calias et al. 2012; Patel et al. 2012; Remeš et al. 2013; Bottros & Christo 2014; Lynch 2014; Lee et al. 2017; Tangen et al. 2019; Fowler et al. 2020; De Andres et al. 2022). Standard ITDD protocols involve either the continuous pumping of the drug through a small catheter or the administration of a finite dose at selected times (Bottros & Christo 2014; Fowler et al. 2020; De Andres et al. 2022), with drug delivery commonly taking place in the lumbar region, as shown in the schematic of figure 1(a). Analgesic delivery via ITDD usually targets sites along the spinal cord close to the injection location, so that reduced drug dispersion is desired, while for other patients there is interest in rapid dispersion towards the cranial cavity, that being the case of intrathecal chemotherapy for brain tumours.

Although ITDD is used with satisfactory results, efforts to optimize the delivery protocol are hindered by the lack of an accurate methodology for predicting drug delivery rates to targeted locations, which sometimes results in unexpected over-dosing and under-dosing complications (Buchser et al. 2004; Wallace & Yaksh 2012) that cannot be explained by existing pharmacokinetics knowledge (Kamran & Wright 2001; Pardridge 2011). The development of predictive models necessitates improved understanding of the interacting convective and diffusive mechanisms controlling the transport of the drug. The present paper, complementing previous computational (Myers 1996; Kuttler et al. 2010; Hsu et al. 2012; Tangen et al. 2015, 2017; Haga et al. 2017; Khani et al. 2018, 2022; Gutiérrez-Montes et al. 2021), experimental (Hettiarachchi et al. 2011; Khani et al. 2022; Seiner et al. 2022; Ayansiji et al. 2023; Moral-Pulido et al. 2023) and theoretical (Sánchez et al. 2018; Lawrence et al. 2019) efforts, seeks to contribute to the needed understanding by analysing effects of buoyancy, which are known by clinicians to play an important role in the dispersion rate of ITDD drugs for patients in an upright or sitting position (Chambers, Edstrom & Scott 1981; Wildsmith et al. 1981; Greene 1985; Hocking & Wildsmith 2004; De Andres et al. 2022). Asymptotic methods based on the disparity of length and time scales present in the problem will be used to derive a reduced transport equation for the drug, enabling accurate predictions of drug dispersion at a fraction of the computational cost associated with direct numerical simulations.

The rest of the paper is organized as follows. After reviewing in § 2 the main features of the flow in the spinal canal, the problem of solute dispersion in the presence of buoyancy forces is formulated in § 3. The asymptotic development leading to the reduced transport equation describing drug dispersion is presented next in § 4. The simplified model is used in § 5 to compute dispersion of positively and negatively buoyant solutes in geometrically simple models of the spinal canal. The results are validated by comparisons with direct numerical simulations, similar to those performed earlier in connection with neutrally buoyant solutes (Gutiérrez-Montes et al. 2021). Computations accounting for anatomically correct spinal canals are presented next, with separate consideration given to drug delivery via bolus injection (§ 6) and constant infusion (§ 7), the latter analysis involving a localized solute source with a rescaled effective Richardson number. Finally, concluding remarks are given in § 8.

## 2. Flow and transport in the spinal canal

The SAS surrounding the spinal cord can be described in the first approximation as a thin annular channel whose characteristic width $h_{c}∼0.1-0.4\;cm$ that is much smaller than the characteristic spinal cord perimeter $\ell_{c}∼2-3\;cm$, which in turn is much smaller than the spine length L∼60cm, so that the canal dimensions satisfy the inequalities $L\gg \ell_{c}\gg h_{c}$. The CSF moves along the canal with an oscillatory velocity that is synchronized with the cardiac and respiratory cycles. The CSF oscillatory flow is more pronounced near the canal entrance, where the characteristic velocities $u_{c}$ are of the order of a few cm s^−1^, but become progressively smaller on approaching the closed end of the canal, as revealed by *in vivo* magnetic resonance measurements (Haughton & Mardal 2014; Aktas et al. 2019; Coenen et al. 2019; Sincomb et al. 2022). The following analysis focuses specifically on the flow induced by the cardiac cycle, corresponding to angular frequencies $\omega ≃2\pi \;s^{-1}$ and characteristic stroke lengths $L_{S}=u_{c}/\omega ∼1\;cm$ that are much smaller than the canal length L.

The motion in the spinal canal is viscous, in that the characteristic viscous time across the canal $h_{c}^{2}/v$ based on the CSF kinematic viscosity $v≃0.7\times 10^{-3}{\;cm}^{2}{\;s}^{-1}$ is comparable to – although somewhat larger than - the characteristic flow oscillation time $\omega^{-1}$, resulting in order-unity values 3≲α≲12 of the Womersley number $\alpha ={\left(h_{c}^{2}\omega /v\right)}^{1/2}$. By way of contrast, effects of inertia associated with convective acceleration are very limited, as measured by the relevant Strouhal number $\omega L/u_{c}=L/L_{s}\gg 1$, the inverse of which defines an asymptotically small parameter $\varepsilon ∼L_{S}/L≃0.02-0.04$. Thus, in the first approximation, the motion in the slender spinal canal is given by a balance between the pressure gradient, the local acceleration and the viscous forces. The resulting linear unsteady lubrication problem can be solved to give closed-form expressions for the leading-order oscillatory velocity (Sánchez et al. 2018; Lawrence et al. 2019), whose time-averaged value is identically zero. Corrections to this solution can be obtained by extending the asymptotic analysis to higher orders in ε≪1 (Sánchez et al. 2018; Lawrence et al. 2019). The first-order velocity corrections, of order $\varepsilon u_{c}$, exhibit non-zero time-averaged values. This steady-streaming velocity, first identified in the seminal computational work of Kuttler et al. (2010), is due partly to the effect of convective acceleration and partly to the canal compliance (see e.g. Bhosale, Parthasarathy & Gazzola (2022a), Bhosale et al. (2022b) and Cui, Bhosale & Gazzola (2024) for recent analyses of steady-streaming flows stemming from boundary compliance). The associated residence times for the bulk flow in the canal $L/\left(\varepsilon u_{c}\right)=\varepsilon^{-2}\omega^{-1}∼30\;min$ are of the order of those observed in *in vivo* experiments employing radioactive tracers to mark the displacement of the CSF particles (Di Chiro 1964; Greitz & Hannerz 1996).

As shown by Lawrence et al. (2019), the disparity between the short time $\omega^{-1}$ characterizing the oscillatory velocity fluctuations and the residence time $\varepsilon^{-2}\omega^{-1}$ associated with the bulk motion can be used in deriving a simplified transport equation for the drug. The analysis revealed that shear-enhanced diffusion (Watson 1983), which is potentially important for solutes with order-unity values of the Schmidt number S=v/κ, is entirely negligible for the large Schmidt numbers S≫1 corresponding to the small molecular diffusivities κ of typical ITDD drugs (e.g. for methotrexate, $\kappa =5.26\times 10^{-10}{\;m}^{2}{\;s}^{-1}$, yielding S≃1330 for $v=0.7\times 10^{-6}{\;m}^{2}{\;s}^{-1}$ ). The evolution of the drug concentration in the long time scale $\varepsilon^{-2}\omega^{-1}$ was found to be governed by a transport equation involving molecular diffusion across the width of the canal and convective transport driven by the time-averaged Lagrangian motion resulting from the combined effects of steady streaming and Stokes drift. The use of this simplified equation effectively circumvents the need to describe the small concentration fluctuations occurring in the short time scale $\omega^{-1}$, thereby drastically reducing computational times. The accuracy and limitations of this time-averaged description have been tested recently by means of comparisons with results of direct numerical simulations spanning hundreds of oscillation cycles (Gutiérrez-Montes et al. 2021), as needed to generate significant dispersion of the solute. The comparisons demonstrate clearly the accuracy of the time-averaged description, which is seen to provide excellent fidelity at a fraction of the computational cost involved in the direct numerical simulations. The present investigation extends our previous analyses of flow and transport in the spinal canal by accounting for the effects of the small density differences between the drug and the CSF. The mathematical development parallels that employed recently in our analysis of buoyant Lagrangian drift in a vertical wavy-walled channel (Alaminos-Quesada et al. 2022).

## 3. Problem description

### 3.1. The Richardson number

As can be seen in table 1, the drug density $\rho_{d}$ of common intrathecal drug solutions is very close to that of the $CSF\left(\rho =1.00059\;g{\;cm}^{-3}\right.$ at 37 °C) (Nicol & Holdcroft 1992; Lui, Polis & Cicutti 1998; McLeod 2004; Hejtmanek, Harvey & Bernards 2011; Lynch 2014). The drug density can be modified by adding different diluents such as saline, glucose and dextrose. Even though the resulting relative differences are very small (i.e. $10^{-4}≲|\rho -\left.\rho_{d}|/\rho ≲10^{-2}\right)$, the associated buoyancy forces affect in a fundamental way the dispersion of the drug. Thus it has been seen that for hyperbaric (i.e. dense) drugs, the transport of the drug is restricted when the patient is seated for some time before moving to a supine position (Mitchell et al. 1988; Povey, Jacobsen & Westergaard-Nielsen 1989; Veering et al. 2001; Loubert et al. 2011). Conversely, when a hypobaric (light) drug is injected, faster cephalic dispersion occurs in a seated injection position than in a lateral injection position (Richardson et al. 1996). As expected, the density of the drug is inconsequential when injection occurs in the lateral position (Hallworth, Fernando & Columb 2005) or when the solution density matches that of CSF (Wildsmith et al. 1981).

To anticipate how the presence of buoyancy forces modifies drug dispersion for patients in a sitting or upright position, it is useful to compare the characteristic value of the buoyancy-induced acceleration $g\left(\rho -\rho_{d}\right)/\rho$ with the characteristic value of the convective acceleration along the canal $u_{c}^{2}/L$, their ratio defining the relevant Richardson number:

(3.1) $$Ri=\frac{g\left(\rho \;-\;\rho_{d}\right)/\rho }{u_{c}^{2}/L}=\frac{g\left(\rho \;-\;\rho_{d}\right)/\rho }{\varepsilon^{2}\omega^{2}L}.$$

Typical values of this number are evaluated in table 1 for a few common intrathecal drugs and two different values of the reduced stroke length ε. As can be seen, values of Ri of order unity characterize most situations of practical interest, so that in ITDD processes, buoyancy acceleration can be anticipated to be comparable to convective acceleration. As discussed previously, the motion of CSF at leading order is given by an unsteady lubrication balance involving the local acceleration and the viscous and pressure forces, with convective acceleration introducing small corrections of order ε, responsible for the steady-streaming motion. This leading-order balance is not altered in the relevant limit Ri∼1 that applies to intrathecal drugs, in which the associated buoyancy-induced velocities are comparable to the steady-streaming velocities (and therefore a factor ε smaller than the pulsating velocities).

### 3.2. The model problem

The problem is formulated in dimensionless form using the scales and notation employed in the previous buoyancy-free analysis of Lawrence et al. (2019), which can be consulted for details of the derivation. Attention is focused on the motion driven by the periodic intracranial pressure fluctuations associated with the arterial blood flow, to be described for simplicity with the simple sinusoidal function $(\Delta p{)}_{c}cos\left(\omega t^{\prime }\right)$, where $(\Delta p{)}_{c}$ is the fluctuation amplitude and $\omega ≃2\pi \;s^{-1}$ is the angular frequency of the cardiac cycle, with $t^{\prime }$ representing the time. The spinal SAS is modelled as an annular canal bounded internally by the pia mater, surrounding the spinal cord, and externally by the dura membrane. The canal is compliant because of the presence of fatty tissue and venous blood. The displacement of the dura membrane at a given location is assumed to be equal to the product of the local pressure fluctuation and a compliance factor $\gamma^{\prime }$ that may vary along the canal. Its mean value $\gamma_{c}^{\prime }$ can be used to estimate the characteristic value of the dura displacement $\gamma_{c}^{\prime }(\Delta p{)}_{c}$, which is much smaller than the canal width, with the ratio

(3.2) $$\varepsilon =\frac{\gamma_{c}^{\prime }(\Delta p{)}_{c}}{h_{c}}∼\frac{L_{s}}{L}$$

defining the small asymptotic parameter representing the dimensionless stroke length.

As indicated in figure 1(c), the problem is described in terms of curvilinear coordinates, including the longitudinal distance to the canal entrance x (scaled with L), the transverse distance from the spinal cord y (scaled with the characteristic canal width $h_{c}$), and the azimuthal distance s (scaled with the local spinal cord perimeter, so that 0⩽s⩽1). The corresponding streamwise, transverse and azimuthal velocity components (u,v,w) are scaled with their characteristic values $u_{c}=\varepsilon \omega L,\;v_{c}=\varepsilon \omega h_{c}$ and $w_{c}=\varepsilon \omega \ell_{c}$, the last two of which follow from continuity. The geometry of the canal is defined by the dimensionless unperturbed canal width $\overset{‾}{h}(x,\;s)$ (scaled with $h_{c}$) and spinal cord perimeter ℓ(x) (scaled with $\ell_{c}$). The linear elastic equation for the canal takes the form

(3.3) $$h^{\prime }=\gamma \left(cos\;t+k^{2}p^{\prime }\right),$$

where $h^{\prime }$ is the dura–membrane displacement (scaled with $\varepsilon h_{c}),\;t=\omega t^{\prime }$ is the dimensionless time, $p^{\prime }(x,\;t)$ is the streamwise pressure variation (scaled with $\rho u_{c}\omega L),\;k=L\omega /{\left[\left(h_{c}/\gamma_{c}^{\prime }\right)/\rho \right]}^{1/2}$ is a dimensionless elastic wavenumber, and $\gamma (x)=\gamma^{\prime }/\gamma_{c}^{\prime }$ is a dimensionless function describing the streamwise variation of the canal compliance.

### 3.3. Dimensionless formulation

In the thin-film approximation that applies in the limit $L\gg \ell_{c}\gg h_{c}$, the continuity, momentum and solute conservation equations take the simplified form

(3.4) $$\frac{1}{\ell }\frac{\partial }{\partial x}(\ell u)+\frac{\partial v}{\partial y}+\frac{1}{\ell }\frac{\partial w}{\partial s}=0,$$

(3.5) $$\frac{\partial u}{\partial t}+\varepsilon \left[u\frac{\partial u}{\partial x}+v\frac{\partial u}{\partial y}+\frac{w}{\ell }\frac{\partial u}{\partial s}\right]=-\frac{\partial p^{\prime }}{\partial x}+\frac{1}{\alpha^{2}}\frac{\partial^{2}u}{\partial y^{2}}-\varepsilon Ric,$$

(3.6) $$\frac{\partial w}{\partial t}+\varepsilon \left[\frac{u}{\ell }\frac{\partial }{\partial x}(\ell w)+v\frac{\partial w}{\partial y}+\frac{w}{\ell }\frac{\partial w}{\partial s}\right]=-\frac{1}{\ell }\frac{\partial \overset{ˆ}{p}}{\partial s}+\frac{1}{\alpha^{2}}\frac{\partial^{2}w}{\partial y^{2}},$$

(3.7) $$\frac{\partial c}{\partial t}+\varepsilon \left(u\frac{\partial c}{\partial x}+v\frac{\partial c}{\partial y}+\frac{w}{\ell }\frac{\partial c}{\partial s}\right)=\frac{\varepsilon^{2}}{\alpha^{2}\sigma }\frac{\partial^{2}c}{\partial y^{2}},$$

where c is the drug concentration and $\overset{ˆ}{p}$ is an auxiliary function describing the azimuthal pressure variations. The problem has been formulated using the Boussinesq approximation, as is appropriate for $\left|\rho -\rho_{d}\right|\ll \rho$. Since the spinal curvature is relatively small, for the case of a sitting patient considered here the streamwise coordinate x is practically aligned with the vertical direction, so that the component of the buoyancy force acting in the azimuthal direction is small, and has been correspondingly neglected in writing (3.6). With the definition (3.1), the Richardson number Ri measuring the buoyancy force in (3.5) is positive/negative when the drug is lighter/heavier than the CSF, buoyancy driving the drug upwards/downwards, in the negative/positive x direction. Following Lawrence et al. (2019), the diffusion term in (3.7) has been written in terms of the reduced Schmidt number $\sigma =\varepsilon^{2}S$, assumed to be of order unity, as is consistent with the values S∼2000 and ε∼0.02−0.04 that characterize drug dispersion in the spinal canal.

The velocity satisfies the no-slip condition u=v=w=0 at y=0, and $u=v-\partial h^{\prime }/\partial t=w=0$ at y=h. Although drug uptake by the spinal nerve as well as through the dura membrane could be incorporated in the model by accounting for non-zero diffusive fluxes at the boundary, for simplicity the following analysis is restricted to non-permeable bounding surfaces, for which the boundary condition for the concentration reduces to ∂c/∂η=0 at y=0,h. The pressure drop is negligible at the entrance of the canal, resulting in the condition $p^{\prime }=0$ at x=0. The requirement that the axial volume flux $\int_{0}^{1}(\int_{0}^{h}u\;dy)ds$ must vanish at the closed end x=1 completes the set of boundary conditions needed to determine the flow in the canal.

Besides the Richardson number Ri defined in (3.1) and the compliance parameter ε≪1 defined in (3.2), the set of governing parameters includes the Womersley number $\alpha =h_{c}/(\nu /\omega {)}^{1/2}$, the dimensionless elastic wavenumber $k=L\omega /{\left[\left(h_{c}/\gamma_{c}^{\prime }\right)/\rho \right]}^{1/2}$, and the rescaled Schmidt number $\sigma =S\varepsilon^{2}$. The problem is to be solved in the limit ε≪1, with α∼1 and k∼1, as is appropriate for describing CSF flow in the spinal canal, for solutes with $\sigma =S\varepsilon^{2}∼1$ and Ri∼1, the distinguished limit of interest in intrathecal drug dispersion.

## 4. Solute transport in the presence of buoyancy

Following our previous analyses (Sánchez et al. 2018; Lawrence et al. 2019; Alaminos-Quesada et al. 2022), the problem defined above is solved by expressing the different variables as expansions in powers of ε (e.g. $u=u_{0}+\varepsilon u_{1}+\cdots$ ) and solving sequentially the equations that arise when collecting terms at different orders in ε. In the development, it is convenient to replace the transverse coordinate y by its normalized counterpart η=y/h, with 0⩽η⩽1. The velocity field depends on the solute concentration through the buoyancy term appearing in (3.5), although the dependence is weak, since ε≪1. The distribution of c can be anticipated to vary over times of the order of the residence time associated with the bulk motion $\varepsilon^{-2}\omega^{-1}$, inducing slow changes in the velocity, to be described below by introducing the long time scale $\tau =\varepsilon^{2}t$ as an additional independent variable. In this two-time scale formalism, all variables are assumed to be 2π periodic in the short time scale t, slow changes in time being described by the additional time variable τ, which is formally introduced in the equations by replacing the original time derivatives by $\partial /\partial t+\varepsilon^{2}\partial /\partial \tau$.

### 4.1. Leading-order solution

At leading order in the limit ε≪1, the problem reduces to the integration of

(4.1) $$\frac{1}{\ell }\frac{\partial }{\partial x}\left(\ell u_{0}\right)-\frac{\eta }{\overset{‾}{h}}\frac{\partial \overset{‾}{h}}{\partial x}\frac{\partial u_{0}}{\partial \eta }+\frac{1}{\overset{‾}{h}}\frac{\partial v_{0}}{\partial \eta }+\frac{1}{\ell }\frac{\partial w_{0}}{\partial s}-\frac{\eta }{\overset{‾}{h}}\frac{1}{\ell }\frac{\partial \overset{‾}{h}}{\partial s}\frac{\partial w_{0}}{\partial \eta }=0,$$

(4.2) $$\frac{\partial u_{0}}{\partial t}=-\frac{\partial p_{0}^{\prime }}{\partial x}+\frac{1}{\alpha^{2}{\overset{‾}{h}}^{2}}\frac{\partial^{2}u_{0}}{\partial \eta^{2}},$$

(4.3) $$\frac{\partial w_{0}}{\partial t}=-\frac{1}{\ell }\frac{\partial {\overset{ˆ}{p}}_{0}}{\partial s}+\frac{1}{\alpha^{2}{\overset{‾}{h}}^{2}}\frac{\partial^{2}w_{0}}{\partial \eta^{2}},$$

(4.4) $$\frac{\partial c_{0}}{\partial t}=0,$$

supplemented with $h_{0}^{\prime }=\gamma (cos\;t+k^{2}p_{0}^{\prime })$, the leading-order form of (3.3), with boundary conditions $u_{0}=v_{0}=w_{0}=\partial c_{0}/\partial \eta =0$ at η=0, and $u_{0}=v_{0}-\partial h_{0}^{\prime }/\partial t=w_{0}=\partial c_{0}/\partial \eta =0$ at $\eta =1,\;p_{0}^{\prime }=0$ at x=0, and $\int_{0}^{1}(\overset{‾}{h}\int_{0}^{1}u_{0}\;d\eta )ds=0$ at x=1. As indicated by (4.4), at leading order the solute concentration varies only in the long time scale τ, variations with the short time scale t affecting only higher-order corrections of relative order ε and smaller. As shown previously (Sánchez et al. 2018), the solution to the periodic linear lubrication problem (4.1)–(4.3) can be written as

(4.5) $$\left.\begin{array}{c} u_{0}=Re\left(i{\;e}^{it}U\right),\;v_{0}=Re\left(i\;e^{it}V\right),\;w_{0}=Re\left({i\;e}^{it}W\right),\\ p_{0}^{\prime }=Re\left(e^{it}P^{\prime }\right),\;{\overset{ˆ}{p}}_{0}=Re\left(e^{it}\overset{ˆ}{P}\right),\;h_{0}^{\prime }=Re\left(e^{it}H^{\prime }\right), \end{array}\right\}$$

where the complex functions U(x,η,s), V(x,η,s), W(x,η,s), $P^{\prime }(x)$, $\overset{ˆ}{P}(x,\;s)$ and $H^{\prime }(x,\;s)$ are given in Appendix A for completeness. The leading-order solution (4.5), identical to that found in our earlier analyses (Sánchez et al. 2018; Lawrence et al. 2019), is buoyancy-free, and therefore independent of the long time scale τ. Buoyancy will be seen to enter at the following order to modify the bulk motion.

### 4.2. Time-averaged Eulerian velocity

While the above harmonic functions (4.5) have zero mean values over an oscillation period, i.e. $\left\langle u_{0}\right\rangle =0$, with $\left\langle \cdot \rangle =\int_{t}^{t+2\pi }\cdot dt/(2\pi \right)$, the velocity corrections $\left(u_{1},v_{1},w_{1}\right)$ contain non-zero cycle-averaged components $\left(\left\langle u_{1}\right\rangle ,\left\langle v_{1}\right\rangle ,\left\langle w_{1}\right\rangle \right)$ that satisfy the quasi-steady conservation equations

(4.6) $$ℱ=\frac{1}{\ell }\frac{\partial }{\partial x}\left(\ell \overset{‾}{h}\left\langle u_{1}\right\rangle \right)+\frac{1}{\ell }\frac{\partial }{\partial s}\left(\overset{‾}{h}\left\langle w_{1}\right\rangle \right)-\frac{\partial }{\partial \eta }\left(\eta \frac{\partial \overset{‾}{h}}{\partial x}\left\langle u_{1}\right\rangle +\frac{\eta }{\ell }\frac{\partial \overset{‾}{h}}{\partial s}\left\langle w_{1}\right\rangle \right)+\frac{\partial \left\langle v_{1}\right\rangle }{\partial \eta },$$

(4.7) $${ℱ}_{x}=-\frac{\partial \left\langle p_{1}^{\prime }\right\rangle }{\partial x}+\frac{1}{{\overset{‾}{h}}^{2}\alpha^{2}}\frac{\partial^{2}\left\langle u_{1}\right\rangle }{\partial \eta^{2}}-Ric_{0},$$

(4.8) $${ℱ}_{s}=-\frac{1}{\ell }\frac{\partial \left\langle {\overset{ˆ}{p}}_{1}\right\rangle }{\partial s}+\frac{1}{{\overset{‾}{h}}^{2}\alpha^{2}}\frac{\partial^{2}\left\langle w_{1}\right\rangle }{\partial \eta^{2}},$$

obtained by taking the time average of the equations that emerge when collecting terms of order ε in (3.4)–(3.6). The functions $ℱ,\;{ℱ}_{x}$ and ${ℱ}_{s}$ appearing on the left-hand side of the above equations carry the combined effects of convective acceleration and canal deformation on the mean Eulerian motion. These functions involve time averages of products of the harmonic functions (4.5), with expressions given in Appendix A.

The velocity must satisfy the homogeneous boundary conditions $\left\langle u_{1}\right\rangle =\left\langle v_{1}\right\rangle =\left\langle w_{1}\right\rangle =0$ at η=(0,1) and $\int_{0}^{1}(\overset{‾}{h}\int_{0}^{1}\left\langle u_{1}\right\rangle d\eta )ds=0$ at x=1. Note that the condition $\left\langle v_{1}\right\rangle =0$ at η=1 follows at this order from the general condition $v=\partial h^{\prime }/\partial t$ written in the two-time scale formalism in the form $v=\partial h^{\prime }/\partial t+\varepsilon^{2}\partial h^{\prime }/\partial \tau$, so that $\langle v\rangle =\varepsilon^{2}\partial \left\langle h^{\prime }\right\rangle /\partial \tau$.

Observation of (4.6)–(4.8) reveals that the mean Eulerian motion has two different driving mechanisms, namely, the buoyancy force $-Ric_{0}$ appearing on the right-hand side of (4.7), which varies slowly in the long time scale τ, and the steady functions $ℱ,\;{ℱ}_{x}$ and ${ℱ}_{s}$, associated with convective acceleration and canal deformation. Since the problem is linear, the two distinct driving mechanisms can be quantified separately by expressing the mean Eulerian velocity $\left(\left\langle u_{1}\right\rangle ,\left\langle v_{1}\right\rangle ,\left\langle w_{1}\right\rangle \right)=\left(u_{SS}+u_{B},v_{SS}+v_{B},w_{SS}+w_{B}\right)$ as the sum of the steady-streaming velocity $\left(u_{SS},v_{SS},w_{SS}\right)$ and the buoyancy-induced drift $\left(u_{B},v_{B},w_{B}\right)$. The former was obtained in our previous analyses (Sánchez et al. 2018; Lawrence et al. 2019) by integration of the problem arising with Ri=0, yielding the solution given in Appendix A, while the latter, the new contribution arising when the drug density differs from the CSF density (i.e. when Ri≠0 ), can be obtained by integration of the reduced problem corresponding to $ℱ={ℱ}_{x}={ℱ}_{s}=0$. The resulting solution, involving integrals of the leading-order solute concentration $c_{0}$, can be cast in the form

(4.9) $$\frac{u_{B}}{\alpha^{2}Ri{\bar{h}}^{2}}=3\eta \left(1\;-\;\eta \right)\frac{\int_{0}^{1}\;{\bar{h}}^{3}𝒞\;ds}{\int_{0}^{1}\;{\bar{h}}^{3}\;ds}+\eta \;\;\int_{0}^{\eta }\;c_{0}\;d\tilde{\eta }-\int_{0}^{\eta }\;c_{0}\tilde{\eta }d\tilde{\eta }-\eta \int_{0}^{1}\;c_{0}\left(1-\eta \right)d\eta ,$$

(4.10) $$\frac{w_{B}}{\alpha^{2}Ri{\overset{‾}{h}}^{2}}=\frac{3\eta (1\;-\;\eta )}{{\overset{‾}{h}}^{3}}\frac{\partial }{\partial x}\left[\ell \left(\int_{0}^{s}{\overset{‾}{h}}^{3}𝒞d\tilde{s}-\frac{\int_{0}^{1}{\overset{‾}{h}}^{3}𝒞\;ds}{\int_{0}^{1}{\overset{‾}{h}}^{3}\;ds}\int_{0}^{s}{\overset{‾}{h}}^{3}\;d\tilde{s}\right)\right],$$

(4.11) $$\frac{v_{B}}{\alpha^{2}Ri}=\frac{\eta^{2}}{\ell }\left(\eta -\frac{3}{2}\right)\frac{\partial }{\partial x}\left(\ell {\overset{‾}{h}}^{3}𝒞\right)-\frac{1}{\ell }\frac{\partial }{\partial x}\left(\ell {\overset{‾}{h}}^{3}f_{B}\right)+\eta \frac{\partial \overset{‾}{h}}{\partial x}\frac{u_{B}}{\alpha^{2}Ri}+\frac{\eta }{\ell }\frac{\partial \overset{‾}{h}}{\partial s}\frac{w_{B}}{\alpha^{2}Ri},$$

where

(4.12) $$𝒞=\int_{0}^{1}c_{0}\eta (1-\eta )d\eta$$

and

(4.13) $$f_{B}=\frac{1}{2}\int_{0}^{\eta }c_{0}{\tilde{\eta }}^{2}\;d\tilde{\eta }+\left(\frac{\eta^{2}}{2}-\eta \right)\int_{0}^{\eta }c_{0}\tilde{\eta }d\tilde{\eta }-\frac{\eta^{2}}{2}\int_{\eta }^{1}c_{0}\left(1-\tilde{\eta }\right)d\tilde{\eta },$$

with tildes used to denote dummy integration variables.

### 4.3. The integro-differential transport equation

As shown by Lawrence et al. (2019), the transport equation that determines the slow spatiotemporal evolution of $c_{0}(x,\;\eta ,\;s,\;\tau )$, given by

(4.14) $$\frac{\partial c_{0}}{\partial \tau }+u_{L}\frac{\partial c_{0}}{\partial x}+\left[\frac{v_{L}}{\overset{‾}{h}}-\frac{\eta }{\overset{‾}{h}}\left(u_{L}\frac{\partial \overset{‾}{h}}{\partial x}+\frac{w_{L}}{\ell }\frac{\partial \overset{‾}{h}}{\partial s}\right)\right]\frac{\partial c_{0}}{\partial \eta }+\frac{w_{L}}{\ell }\frac{\partial c_{0}}{\partial s}=\frac{1}{\alpha^{2}\sigma {\overset{‾}{h}}^{2}}\frac{\partial^{2}c_{0}}{\partial \eta^{2}},$$

can be obtained by analysing terms of order $\varepsilon^{2}$ in (3.7). The convective transport in the long time scale is found to be driven by the mean Lagrangian velocity

(4.15) $$\left.\begin{array}{l} u_{L}=u_{SS}+u_{B}+u_{SD},\\ v_{L}=v_{SS}+v_{B}+v_{SD},\\ w_{L}=w_{SS}+w_{B}+w_{SD}, \end{array}\right\}$$

given by the sum of the cycle-averaged Eulerian velocity $\left(\left\langle u_{1}\right\rangle ,\left\langle v_{1}\right\rangle ,\left\langle w_{1}\right\rangle \right)=\left(u_{SS}+\right.\left.{\;u}_{B},v_{SS}+v_{B},w_{SS}+w_{B}\right)$ and the Stokes drift $\left(u_{SD},v_{SD},w_{SD}\right)$, the latter being a purely kinematic contribution resulting from the spatial non-uniformity of the pulsatile flow (Lawrence et al. 2019). The steady-streaming and Stokes-drift contributions to the time-averaged Lagrangian motion, constant and independent of the drug concentration, were identified in our previous analysis (Lawrence et al. 2019), with corresponding expressions given in Appendix A. The slowly varying buoyancy-induced velocity $\left(u_{B},v_{B},w_{B}\right)$ is a new contribution coupling the bulk motion with the drug concentration. Since the expressions for $\left(u_{B},v_{B},w_{B}\right)$, given in (4.9)–(4.11), contain spatial integrals of the solute concentration $c_{0}$, the transport equation (4.14), which is a linear partial differential equation in the buoyancy-free case Ri=0 analysed earlier (Lawrence et al. 2019), adopts for Ri≠0 a nonlinear integro-differential character that complicates the description.

The transport equation (4.14), supplemented with (4.9)–(4.11) for the evaluation of the slowly varying buoyancy-induced velocity $\left(u_{B},v_{B},w_{B}\right)$ and with the expressions given in Appendix A for the time-independent velocity components ($u_{SS},v_{SS},w_{SS}$) and $\left(u_{SD},v_{SD},w_{SD}\right)$, can be integrated with boundary conditions $\partial c_{0}/\partial \eta =0$ at η=(0,1) to determine the evolution of the solute. An additional condition must be prescribed at points across the entrance section x=0 where there exists inflow (i.e. positive values of $u_{L}$ ). In the following integrations, it is assumed that the drug concentration of the incoming fluid particles is identically zero, as is consistent with drug delivery in the lumbar region. Bolus injection can be described by using as initial condition the solute distribution $c_{0}=c_{i}(x,\;\eta ,\;s)$ existing at the end of the short injection phase. The description of continuous drug infusion is somewhat more complicated, in that it requires consideration of a localized solute source at the delivery location, a case to be addressed separately in § 7.

Although the reduced Schmidt number $\sigma =S\varepsilon^{2}$ can be expected to take order-unity values for the drugs typically used in applications (e.g. σ=0.532−2.128 when evaluated with ε=0.02−0.04 for methotrexate), it is instructive to investigate simplifications arising for extreme values of this parameter. For example, for σ≫1, the transverse-diffusion term in (4.14) becomes negligible, with the result that the solute particles are transported by the mean Lagrangian velocity while maintaining its initial concentration. Numerical methods specifically tailored to describe Lagrangian particle dispersion can be instrumental to speed up the associated computations (Guan et al. 2023). In the opposite limit, σ≪1, diffusion rapidly uniformizes the composition in the transverse direction, so that the concentration becomes independent of η. The simplified equation applying in this limit can be derived by integrating (4.14) in η with boundary conditions $\partial c_{0}/\partial \eta =0$ at η=(0,1), to yield

(4.16) $$\frac{\partial c_{0}}{\partial \tau }+{\overset{‾}{u}}_{L}\frac{\partial c_{0}}{\partial x}+\frac{{\overset{‾}{w}}_{L}}{\ell }\frac{\partial c_{0}}{\partial s}=0,$$

where ${\overset{‾}{u}}_{L}=\int_{0}^{1}u_{L}\;d\eta$ and ${\overset{‾}{w}}_{L}=\int_{0}^{1}w_{L}\;d\eta$ are the width-averaged values of the longitudinal and azimuthal components of the mean Lagrangian velocity. It will be of interest in future work to assess the predictive capability of the above simple equation.

It is worth noting that, unlike direct numerical simulations (DNS) of drug delivery, which need to account for the small cumulative concentration changes that occur over subsequent cardiac cycles, the reduced description (4.14) targets directly the solute evolution in the long time scale $\varepsilon^{-2}\omega^{-1}$ that characterizes drug dispersion along the canal. Since the number of cardiac cycles required to achieve significant drug dispersion scales with $\varepsilon^{-2}$, DNS computations accounting for realistic values of ε∼0.02−0.04 must in general consider hundreds of cycles, resulting in computational times that are orders of magnitude larger than those involved in integrating (4.14).

## 5. Validation of the reduced model

For buoyancy-free systems (i.e. Ri=0), the mean Lagrangian velocity reduces to $\left(u_{L},v_{L},w_{L}\right)=\left(u_{SS}+u_{SD},v_{SS}+v_{SD},w_{SS}+w_{SD}\right),\;$ independent of the solute concentration, with the result that the associated transport equation (4.14) becomes a linear partial differential equation with time-independent coefficients. The accuracy of the resulting simplified description was tested previously (Gutiérrez-Montes et al. 2021) by comparing the model predictions with results of DNS computations involving integrations of the complete Navier–Stokes equations. The previous comparisons are extended here to cases with Ri≠0, for which (4.14) displays its complicated nonlinear integro-differential character. As in the previous paper, results are given below for two different geometrical configurations with constant perimeter ℓ=1, namely, a constant-eccentricity annular canal bounded by parallel cylindrical surfaces, yielding a canal width $\overset{‾}{h}\left(s\right)=1-0.5\;cos\;(2\pi s)$, and a variable-eccentricity configuration with canal width $\overset{‾}{h}\left(x,\;s\right)=1-0.5\cos\left(2\pi s\right)cos\;(2\pi x)$. The latter geometry is selected as a simplified model to mimic changes in the position of the spinal cord relative to the dura mater existing along the human spinal canal, which are depicted in figures 1(b) and 1(c). As one traverses the spine caudally, the spinal cord, which is closer to the posterior side of the canal in the cervical region, moves closer to the anterior side in the thoracic region, eventually returning to the posterior side in the lumbar region. These changes in the spinal canal eccentricity are known to produce changes in the direction of the longitudinal mean Lagrangian velocity (Coenen et al. 2019), leading to the recirculating pattern of bulk CSF flow shown in figure 1(d).

The validation addresses the temporal evolution of the solute following the release of a finite dose, with the initial solute concentration described by the truncated Gaussian distribution

(5.1) $$c_{i}=min\left\{1,\;\frac{3}{2}exp\left[-16{\left(\frac{x\;-\;x_{0}}{\delta }\right)}^{2}\right]\right\},$$

which represents a band of solute with characteristic width δ centred at $x_{0}$ and having a saturated core flanked by thin layers across which the concentration decays to zero. The values δ=0.2 and $x_{0}=0.65$ are selected in the sample computations shown below.

The numerical scheme for the integration of (4.14) utilizes a second-order centred finite-difference approximation for the spatial discretization of the viscous terms, and an upwind scheme for the nonlinear terms. A second-order explicit Runge–Kutta scheme is used for time marching, with the integral expressions (4.9)–(4.11) evaluated with a simple trapezoidal rule. A detailed account of the numerical scheme employed in the accompanying DNS computations can be found in Gutiérrez-Montes et al. (2021). The DNS computations were performed for a dimensionless stroke length ε=0.02, so that every unit in the long time scale τ corresponds to $(2\pi \varepsilon {)}^{-2}≃400$ oscillatory cycles in the DNS computations. The resulting concentration, which includes short-time fluctuations associated with the oscillatory flow, is cycled-averaged to give $\left\langle c\rangle =\int_{t}^{t+2\pi }c\;dt/(2\pi \right)$, to be compared with the associated model prediction $c_{0}$.

**Figure 2.**
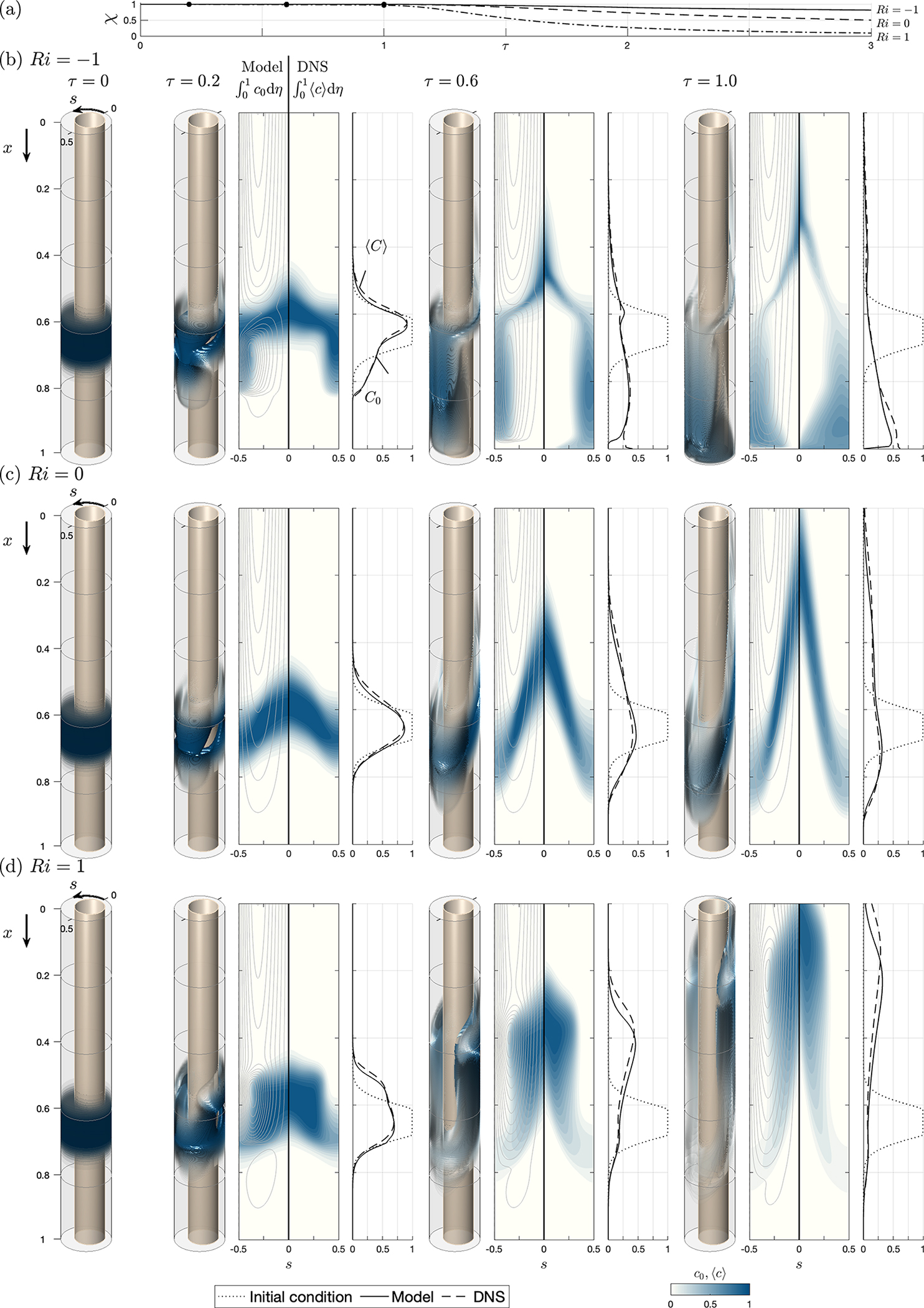
The temporal evolution of the solute concentration in a constant-eccentricity canal with $\ell =1,\;\overset{‾}{h}\left(s\right)=1-0.5\;cos\;(2\pi s)$, α=3,k=0.5,γ=1 and σ=0.4 as obtained from the reduced transport equation (4.14) and from DNS computations for three different values of the Richardson number, (*b*) Ri=−1, (*c*) Ri=0 and (*d*) Ri=1, with (a) showing the temporal evolution of the total amount of solute contained in the canal (normalized with its initial value) predicted with the reduced model, as computed from $\chi =\int_{0}^{1}C_{0}\;dx/\int_{0}^{1}C_{i}\;dx$. The plots include three-dimensional isosurfaces of solute concentration $c_{0}$, distributions of width-averaged concentrations $\int_{0}^{1}c_{0}\;d\eta$ and $\int_{0}^{1}\langle c\rangle d\eta$, and corresponding axial distributions of concentration per unit length of canal $C_{0}=\int_{0}^{1}\overset{‾}{h}\int_{0}^{1}c_{0}\;d\eta \;ds$ (solid curves) and $\langle C\rangle =\int_{0}^{1}\overset{‾}{h}\int_{0}^{1}\langle c\rangle d\eta \;ds$ (dashed curves), with the dotted curves representing the initial distribution $C_{i}=\int_{0}^{1}\overset{‾}{h}\int_{0}^{1}c_{i}\;d\eta \;ds$. The streamlines shown in the plots of $\int_{0}^{1}c_{0}\;d\eta$, corresponding to the width-averaged Lagrangian drift velocity $\left(\int_{0}^{1}u_{L}\;d\eta ,\int_{0}^{1}w_{L}\;d\eta \right)$, are plotted using constant spacing 0.01 for the associated width-averaged stream function.

**Figure 3.**
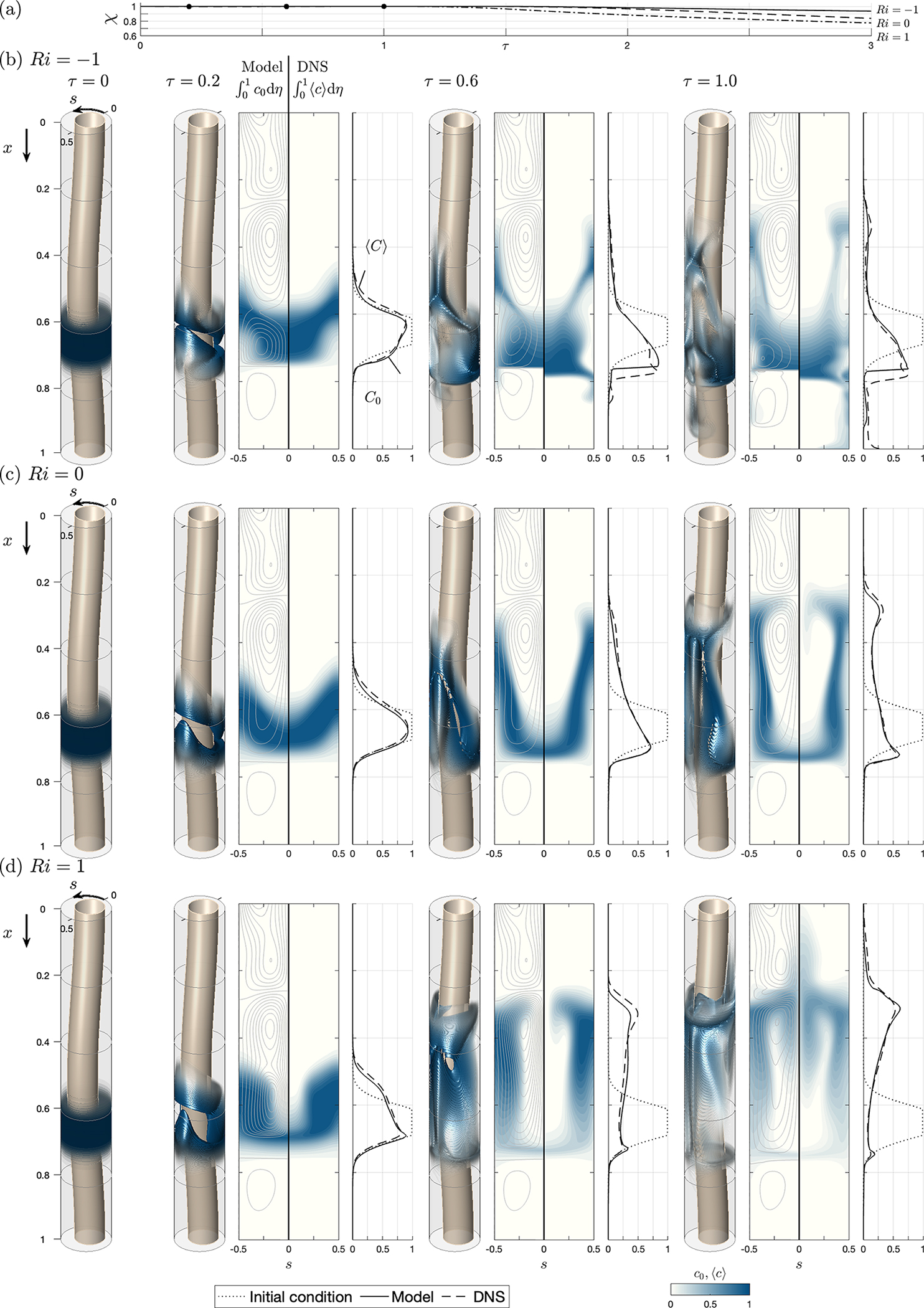
Same as figure 2 but for a variable eccentricity canal with $\overset{‾}{h}\left(x,\;s\right)=1-0.5\cos\left(2\pi s\right)cos\;(2\pi x)$.

Results are shown in figures 2 (constant eccentricity) and 3 (variable eccentricity) for a canal with α=3,k=0.5,γ=1 and σ=0.4. To illustrate effects of buoyancy on drug dispersion, in addition to the buoyancy-neutral case Ri=0, the computations consider both a heavy solute with $\rho_{d}>\rho \;(Ri=-1)$ and a light solute with $\rho_{d}<\rho \;(Ri=1)$. The figures display three-dimensional views of the entire canal showing isosurfaces of solute concentration $c_{0}$ for several values of τ. The quantitative comparisons between the model and the DNS include distributions of width-averaged concentrations $\int_{0}^{1}c_{0}\;d\eta$ and $\int_{0}^{1}\left\langle c\right\rangle d\eta$ as well as corresponding axial distributions of concentration per unit length of canal, computed according to $C_{0}=\int_{0}^{1}\overset{‾}{h}\int_{0}^{1}c_{0}\;d\eta \;ds$ and $\langle C\rangle =\int_{0}^{1}\overset{‾}{h}\int_{0}^{1}\langle c\rangle d\eta \;ds$, with the dotted curves representing the initial distribution $C_{i}=\int_{0}^{1}\overset{‾}{h}\int_{0}^{1}c_{i}\;d\eta \;ds$. For reference, the left-hand contour panels showing $\int_{0}^{1}c_{0}\;d\eta$ include the streamlines corresponding to the width-averaged Lagrangian drift velocity $\left(\int_{0}^{1}u_{L}\;d\eta ,\int_{0}^{1}w_{L}\;d\eta \right)$, which evolve in time under the action of buoyancy when Ri≠0. Figures 2(a) and 3(a) indicate the fraction of the drug bolus that remains in the canal at time τ, as computed with the reduced transport model according to $\chi =\int_{0}^{1}C_{0}\;dx/\int_{0}^{1}C_{i}\;dx$.

Observation of the plots displaying streamlines reveals that the solute moves predominantly following the width-averaged flow, thereby highlighting the important role of the Lagrangian drift in the dispersion of the drug. For a non-buoyant solute in a constant-eccentricity canal, investigated in figure 2(c), the mean Lagrangian flow exhibits a simple circulating pattern, in which the fluid enters along the wide part of the canal (s=0.5) and leaves along the narrow part (s=0), the motion being slower near the closed end x=1. As seen in figures 2(b) and 2(d), the presence of buoyancy alters the flow, with associated streamlines evolving in time as the spatial distribution of the solute changes. Buoyancy promotes rapid ascension of the light solute along the narrow part of the canal, that being the behaviour displayed in figure 2(d). Conversely, heavy solutes tend to sink to the bottom, progression towards the canal entrance being limited to a thin solute filament stretching along the narrow section s=0, as seen in figure 2(b). While the overall agreement between the model and the DNS is generally satisfactory, a notable deviation arises at x=1 in the heavy-solute results. Here, the model predicts a zero concentration for all times, whereas the DNS yield a concentration that increases over time. These disparities stem from the effect of axial diffusion (not present in the model), which, though negligible elsewhere, becomes significant in this terminal region as the velocity diminishes to zero.

Buoyancy effects are clearly visible in the axial distributions of concentration per unit length of canal $C_{0}$ and ⟨C⟩, and also in the curves representing in figure 2(a) the fraction χ of the initial bolus that remains inside the canal at time τ. The results indicate that at the longest time computed (τ=3), most of the light solute (91 %) has abandoned the canal, while approximately 82% of the heavy solute remains inside. This behaviour is consistent with previous clinical observations pertaining to hyperbaric and hypobaric drugs (Mitchell et al. 1988; Povey et al. 1989; Richardson et al. 1996; Veering et al. 2001; Loubert et al. 2011).

For the variable-eccentricity canal shown in figure 3, the streamline patterns of the mean Lagrangian motion feature multiple recirculating regions. The flow direction is reversed between contiguous recirculating cells, as can be inferred from the maps of solute concentration. The solute, carried by the fluid particles, encircles the recirculating regions, thereby hindering the solute progression towards the canal entrance. The plots at τ=1 show most of the light solute accumulating at the interface separating near x=0.25 the two top recirculating regions (see figure 3d), while the heavy solute accumulates around x=0.75, above the nearly stagnant bottom recirculating region, as shown in figure 3(b). As indicated by comparison of figures 2(a) and 3(a), the rate at which the solute reaches the canal entrance is significantly lower for canals with variable eccentricity, in accordance with previous results (Coenen et al. 2019; Gutiérrez-Montes et al. 2021).

The agreement between the model and the DNS results is very satisfactory, quantitative departures remaining consistently small regardless of the value of Ri. The degree of agreement is particularly remarkable in connection with the dashed and solid curves representing the longitudinal distribution of the solute at different instants of time. In view of the comparisons shown in figures 2 and 3, it can be concluded that the reduced model provides a sufficiently accurate description for most purposes while requiring computational times that are a fraction of those involved in the DNS computations. For instance, to generate the results corresponding to each value of Ri in figures 2 and 3, the computations using the reduced model were completed in approximately 10 minutes using a laptop computer, whereas the DNS computations took approximately a week on a 24 -core cluster.

## 6. Dispersion of a drug bolus

The reduced transport equation (4.14) can be used to generate predictions of drug dispersion based on subject-specific canal boundaries and dimensions, with the model parameters determined using magnetic resonance imaging (MRI) measurements, as explained in Coenen et al. (2019). The sample computations shown below use measurements corresponding to a 25-year old woman (subject 1 in Coenen et al. 2019), with relevant anatomical and Lagrangian-flow details shown in figures 1(b)–1(d). High-resolution images of the entire spine were segmented to extract the three-dimensional position of the pia and dura mater, with the cauda equina (the group of roots branching off at the end of the spinal cord in the lumbar region) represented as an extension of the spinal cord with cross-sectional area tapering down to the end of the spinal canal. The resulting canal anatomy is shown in figure 1(c), with the transverse dimension scaled by a factor 3 to facilitate visualization. A Gaussian filter was used to generate smooth distributions of the perimeter and width of the canal, their mean values $\ell_{c}=21.8\;mm$ and $h_{c}=3.6\;mm$ employed to scale the geometrical functions ℓ(x) and $\overset{‾}{h}(x,\;s)$ used in the model, with the longitudinal distance x being scaled with the total canal length L=59cm. As explained in Coenen et al. (2019), the compliance of the canal was determined by comparing predictions of oscillatory flow rate with phase-contrast MRI measurements, yielding the function $\gamma^{\prime }\left(x\right)=14.3\left[0.8+0.3\tanh\left(4x-0.2\right)\right]m\;{MPa}^{-1}$ with mean value $\gamma_{c}^{\prime }=14.107\;m{\;MPa}^{-1}$. For this subject, the associated values of the Womersley number and elastic wavenumber were found to be $\alpha =h_{c}/(\nu /\omega {)}^{1/2}=10.8$ and $k=L\omega /{\left[\left(h_{c}/\gamma_{c}^{\prime }\right)/\rho \right]}^{1/2}=0.73$, respectively.

**Figure 4.**
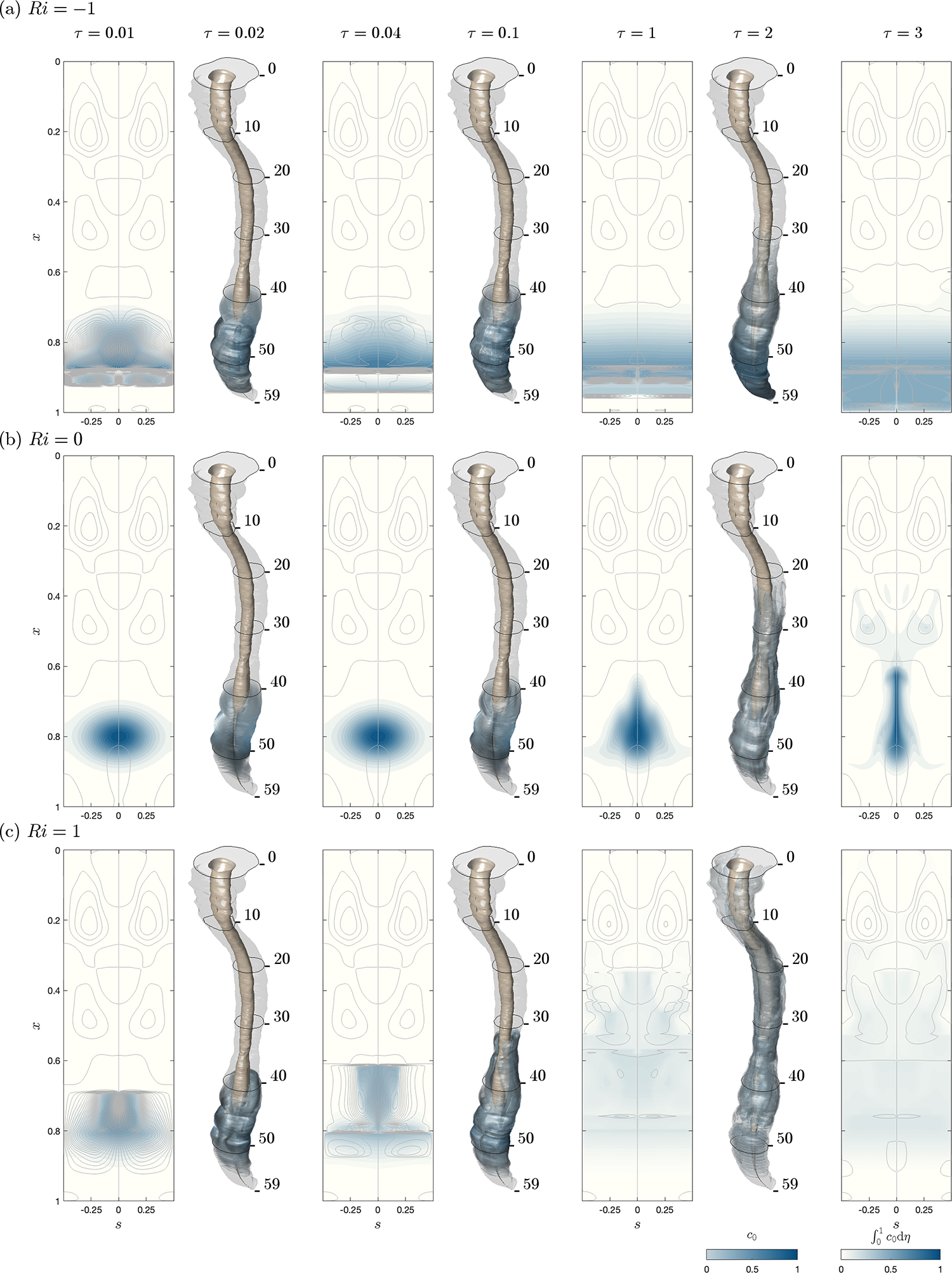
Drug dispersion following delivery of a finite dose via the L3/L4 intervertebral space as predicted for σ=1 and three different values of the Richardson number, (*a*) Ri=−1, (*b*) Ri=0 and (*c*) Ri=1, by integration of the reduced transport equation (4.14) subject to the initial condition (6.1). The plots include distributions of width-averaged concentrations $\int_{0}^{1}c_{0}\;d\eta$ at τ=0.01,0.04,1,3 along with three-dimensional isosurfaces of solute concentration $c_{0}$ at intermediate times τ=0.02,0.1,2.

As discussed earlier in connection with figures 2 and 3, the solute moves predominantly following the Lagrangian drift. Before computing drug dispersion, it is therefore of interest to investigate the structure of the mean Lagrangian flow in the absence of buoyancy forces for the anatomically correct canal shown in figure 1(c). To that end, streamlines corresponding to the width-averaged velocity $\left(\int_{0}^{1}u_{L}\;d\eta ,\int_{0}^{1}w_{L}\;d\eta \right)$ with $\left(u_{L},w_{L}\right)=\left(u_{SS}+u_{SD},w_{SS}+w_{SD}\right)$ are plotted in figure 1(d). The resulting flow pattern comprises three main recirculating regions that occupy approximately the cervical, thoracic and lumbar regions, along with smaller recirculating regions distributed along the posterior midline (s=0). The streamlines plotted correspond to evenly spaced values of the associated stream function, so that the physical distance between contiguous streamlines is a measure of the local flow velocity. As is clear from the plot, the fluid is nearly stagnant in the lumbar region, where drug delivery usually takes place, suggesting that neutrally buoyant or heavy drugs will tend to remain near the injection site. The extent to which buoyancy promotes the dispersion of light drugs is to be evaluated in figure 4(c).

To mimic an intrathecal injection via the L3/L4 posterior intervertebral space, the description of drug dispersion utilizes as initial condition the Gaussian solute distribution

(6.1) $$c_{i}=\exp\left\{-\left[{\left(\frac{x\;-\;x_{0}}{\delta_{x}}\right)}^{2}+{\left(\frac{\eta \;-\;\eta_{0}}{\delta_{\eta }}\right)}^{2}+{\left(\frac{s\;-\;s_{0}}{\delta_{s}}\right)}^{2}\right]\right\},$$

with $\left(x_{0},\eta_{0},s_{0}\right)=(0.8,\;0.5,\;0)$ and $\left(\delta_{x},\delta_{\eta },\delta_{s}\right)=(1/16,\;500,\;2/7)$. The reduced Schmidt number is selected to be $\sigma =\varepsilon^{2}S=1$, corresponding to a drug Schmidt number in the range 625<S<2500 for ε=0.02−0.04. Buoyancy effects are investigated for Ri=1 and −1, taken as representative of Midazolam and Morphine. Their temporal evolution is compared in figure 4 with results corresponding to a neutrally buoyant drug. To facilitate visualization, besides three-dimensional distributions of drug concentration $c_{0}$, the figure shows two-dimensional maps of width-averaged concentration $\int_{0}^{1}c_{0}\;d\eta$ at selected times, with particular attention given to the short time evolution. For the three cases considered, corresponding supplementary movies are available at https://doi.org/10.1017/jfm.2024.297, showing the evolution of the drug up to τ=5.

The plots in figure 4(b) reveal that since the mean Lagrangian motion exhibits low velocities in the lumbar region, in the absence of buoyancy the initial drug evolution is very slow, with changes in the solute concentration distribution remaining virtually inappreciable for τ⩽0.1. For longer times, the drug spreads following the lumbar recirculating vortices, with the result that the drug concentrates in an elongated region about the s=0 axis. For the longest time shown in the figure (τ=3), only a small amount of drug has moved into the thoracic region.

Buoyancy fundamentally alters this dispersion pattern, as seen in figures 4(a) and 4(c). For the localized drug distribution considered in the computations, a fast buoyancy-driven vortex is formed upon injection, as revealed by the closely spaced streamlines shown in the two-dimensional plots for τ=0.01 and 0.04, rapidly spreading the drug around the spinal cord from the initial injection site. The associated recirculatory motion is directed upwards/downwards along the s=0 axis for a light/heavy drug, thereby promoting drug dispersion towards the cranial cavity/sacrum region. The progression rate, very rapid for short times, when the buoyancy-induced velocities are larger as a result of the existing high solute concentrations, slows down for longer times, with the heavy drug adopting a stratified distribution that slowly sinks towards the bottom end of the canal, while the light drug continues to evolve upwards, spreading through the thoracic and cervical regions, and eventually reaching the cranial cavity. The behaviour revealed in the figure is therefore consistent with clinical observations regarding intrathecal injections in a seated position (Wildsmith et al. 1981; Mitchell et al. 1988; Povey et al. 1989; Richardson et al. 1996; Veering et al. 2001).

## 7. The description of continuous drug infusion

Medication by ITDD is often released by continuous infusion with use of a percutaneous catheter connected to an external pump or a totally implanted system. The delivery rates are usually small, with maximum values $\dot{Q}≲1\;ml\;h^{-1}$ (De Andres et al. 2022). Since drug dispersion is driven by the mean Lagrangian motion, it can be anticipated that the total volume of drug released in times of order of the characteristic bulk flow residence time $\varepsilon^{-2}\omega^{-1}$, given by $\dot{Q}\varepsilon^{-2}\omega^{-1}$, will be spread over the entire volume of the canal, $L\ell_{c}h_{c}∼40-60ml$, resulting in characteristic drug concentrations of order

(7.1) $$c_{c}=\frac{\dot{Q}\varepsilon^{-2}\omega^{-1}}{L\ell_{c}h_{c}},$$

with $c_{c}≲0.01$. As a result, in describing continuous drug infusion, it is appropriate to use an order-unity rescaled concentration $\varphi =c/c_{c}$. Also, since the density differences associated with the presence of the drug can be expected to be of order $c_{c}\left(\rho -\rho_{d}\right)$, the Richardson number (3.1), which was defined assuming solute concentrations of order unity, must be replaced with

(7.2) $$Ri^{*}=\frac{g\left(\rho \;-\;\rho_{d}\right)c_{c}/\rho }{\varepsilon^{2}\omega^{2}L},$$

so that the buoyancy acceleration term −εRic in (3.5) becomes $-\varepsilon \;Ri^{*}\varphi$.

Drug injection will be modelled using a localized volume source. To evaluate the contribution of the source to the mass and momentum balance, we must compare the characteristic value of the velocity induced by the source $\dot{Q}/\left(\ell_{c}h_{c}\right)$, obtained by dividing the volumetric injection rate $\dot{Q}$ by the characteristic canal cross-section $\ell_{c}h_{c}$, with the characteristic bulk flow velocity $\varepsilon^{2}\omega L$, the ratio of both quantities reducing simply to $\left[\dot{Q}/\left(\ell_{c}h_{c}\right)\right]/\left(\varepsilon^{2}\omega L\right)=c_{c}\ll 1$, as can be seen from (7.1). Since drug infusion induces negligibly small velocities, the presence of the localized source can be neglected in the first approximation when writing the continuity and momentum balance equations (3.4)–(3.6), but not in the solute conservation equation (3.7), which takes the form

(7.3) $$\frac{\partial \varphi }{\partial t}+\varepsilon \left(u\frac{\partial \varphi }{\partial x}+v\frac{\partial \varphi }{\partial y}+\frac{w}{\ell }\frac{\partial \varphi }{\partial s}\right)=\frac{\varepsilon^{2}}{\alpha^{2}\sigma }\frac{\partial^{2}\varphi }{\partial y^{2}}+\varepsilon^{2}q,$$

where the dimensionless function q(x,η,s) represents the delivery rate per unit volume, scaled with $\dot{Q}/\left(L\ell_{c}h_{c}\right)$, so that $\int_{0}^{1}\ell \int_{0}^{1}\overset{‾}{h}\int_{0}^{1}q\;d\eta \;ds\;dx=1$. The asymptotic analysis, which parallels that leading to (4.14), provides in this case the reduced transport equation

(7.4) $$\frac{\partial \varphi_{0}}{\partial \tau }+u_{L}\frac{\partial \varphi_{0}}{\partial x}+\left[\frac{v_{L}}{\overset{‾}{h}}-\frac{\eta }{\overset{‾}{h}}\left(u_{L}\frac{\partial \overset{‾}{h}}{\partial x}+\frac{w_{L}}{\ell }\frac{\partial \overset{‾}{h}}{\partial s}\right)\right]\frac{\partial \varphi_{0}}{\partial \eta }+\frac{w_{L}}{\ell }\frac{\partial \varphi_{0}}{\partial s}=\frac{1}{\alpha^{2}\sigma {\overset{‾}{h}}^{2}}\frac{\partial^{2}\varphi_{0}}{\partial \eta^{2}}+q$$

for the leading-order representation $\varphi_{0}$ of the reduced solute concentration $\varphi =\varphi_{0}+\varepsilon \varphi_{1}+\cdots$, with the buoyancy-driven component $\left(u_{B},v_{B},w_{B}\right)$ of the Lagrangian drift velocity $\left(u_{L},v_{L},w_{L}\right)$ evaluated from (4.9)–(4.11), with Ri and $c_{0}$ replaced by $Ri^{*}$ and $\varphi_{0}$.

**Figure 5.**
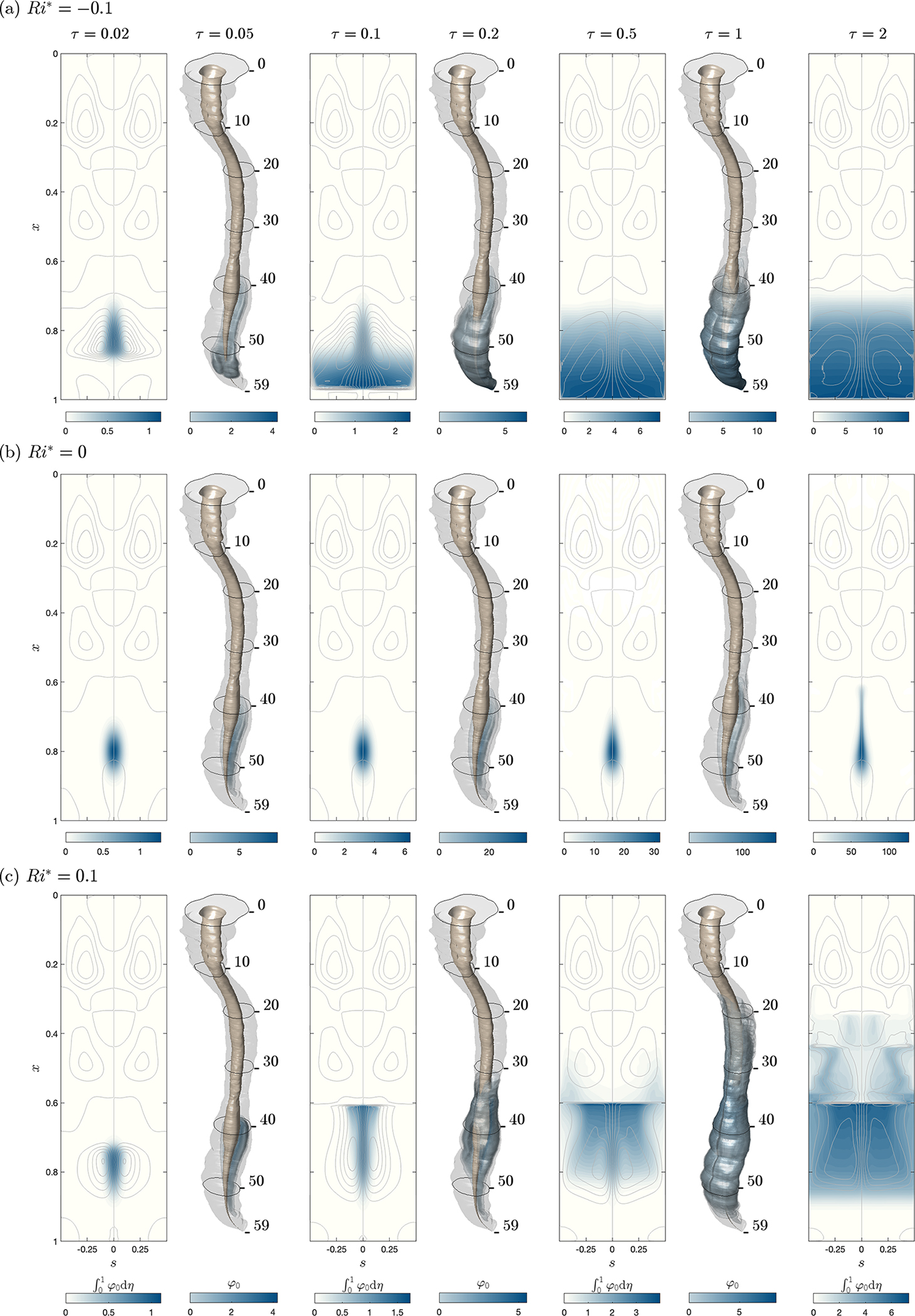
Drug dispersion corresponding to continuous drug infusion via the L3/L4 intervertebral space as predicted for σ=1 and three different values of the rescaled Richardson number, $(a)Ri^{*}=-0.1$, (*b*) $Ri^{*}=0$ and (c) $Ri^{*}=0.1$, by integration of the reduced transport equation (7.4) with a localized solute source centred at $\left(x_{0},\eta_{0},s_{0}\right)=(0.8,\;0.5,\;0)$. The plots include distributions of width-averaged concentrations $\int_{0}^{1}\varphi_{0}\;d\eta$ at τ=0.02,0.1,0.5,2 along with three-dimensional isosurfaces of solute concentration $\varphi_{0}$ at intermediate times τ=0.05,0.2,1.

To represent injection in the posterior intrathecal region through the L3/L4 intervertebral space, the sample computations shown in figure 5 consider a localized source with a normalized Gaussian distribution $q(x,\;\eta ,\;s)=q_{o}/(\int_{0}^{1}\ell \int_{0}^{1}\overset{‾}{h}\int_{0}^{1}q_{o}\;d\eta \;ds\;dx)$ centred at $\left(x_{0},\eta_{0},s_{0}\right)=(0.8,\;0.5,\;0)$, where the function $q_{o}$ is the exponential distribution found on the right-hand side of (6.1) with $\left(\delta_{x},\delta_{\eta },\delta_{s}\right)=(1/18,\;1/5,\;1/13)$. For the three cases considered, corresponding supplementary movies are available. The integrations, initiated with a zero drug concentration everywhere in the canal, describe transient drug infusion for three different reduced Richardson numbers $Ri^{*}=c_{c}Ri$, with the values $Ri^{*}=-0.1$ and 0.1 being comparable to, although somewhat larger than, those expected in connection with the dispersion of Meperidine and Fentanyl (see table 1). As in figure 4, figure 5 shows three-dimensional distributions of drug concentration $\varphi_{0}$ along with two-dimensional maps of width-averaged concentration $\int_{0}^{1}\varphi_{0}\;d\eta$. Note that for each plot, the scale of the colour contours has been adjusted to accommodate the increasing concentration, which is found to be significantly larger for non-buoyant drugs.

As can be seen in the plots of figure 5(b), the neutrally buoyant drug accumulates near the injection location while spreading longitudinally along the posterior axis s=0 at a small rate determined by the existing mean Lagrangian velocity. In contrast, the heavy drug with $Ri^{*}=-0.1$, shown in figure 5(a), immediately begins to sink upon injection, driving a recirculatory motion that promotes simultaneous azimuthal spreading. At τ=0.2, the drug has already reached the sacral end of the canal, where it accumulates, forming a stratified distribution that is continuously stirred by the persistent buoyancy-driven recirculatory flow. Up to the longest time considered (τ=2), the heavy drug is confined to the lumbar region, with the result that the mean Lagrangian motion remains virtually unperturbed in the thoracic and cervical regions. On the other hand, infusion of light drugs, considered in figure 5(c), leads to the development of a plume. The light fluid rises until it reaches the boundary separating the lumbar and thoracic recirculating regions, forming a front at x≃0.6, corresponding approximately to the T11/T12 intervertebral space. At that level, the drug spreads azimuthally to reach the anterior side, where it continues to flow upwards into the thoracic region, thereby resuming its progression towards the cranial cavity.

In analysing the transient results of figure 5, one should bear in mind that while the present computation assumes impermeable surfaces, leading to continuous drug accumulation, in ITDD processes drug uptake by the spinal nerve as well as through the dura membrane would eventually balance the infusion rate, leading to a steady drug distribution along the spine. For heavy drugs, the results shown in figure 5(a) suggest that the combined effects of buoyancy forces and drug uptake may limit drug dispersion to the lumbar and sacral regions. On the other hand, the results in figure 5(c) indicate that the ability of light drugs to reach the cranial cavity will depend on the competition of buoyancy-enhanced drug dispersion and drug absorption, whose quantification necessitates an extended reduced model accounting for pharmacokinetic effects.

## 8. Conclusions

Asymptotic and numerical methods have been used to quantify, for the first time, effects of buoyancy on the dispersion of drugs delivered in the spinal intrathecal space. A two-time scale asymptotic analysis, similar to that employed in a recent investigation pertaining to a wavy-walled planar channel (Alaminos-Quesada et al. 2022), leads to a simplified transport description targeting the relevant long time scale characterizing drug dispersion.

Since the buoyancy-driven component of the mean Lagrangian velocity driving the convective transport depends on spatial integrals of the solute concentration, as described in (4.9)–(4.11), the resulting solute transport equation, given in (4.14), displays an integro-differential character. The accuracy of the model is tested in computations of buoyancy-modulated solute dispersion in constant-eccentricity and variable-eccentricity annular canals. The model predictions are shown in figures 2 and 3 to be in excellent quantitative agreement with DNS results for positively, neutrally and negatively buoyant solutes, with the computational cost associated with integrations of the reduced transport equation typically being three to four orders of magnitude smaller than those involved in the DNS computations. It is worth mentioning that the two-time scale methodology developed here can find application in analysing buoyancy-modulated secondary motion in other applications involving small density differences, including those related to active particles (Guan et al. 2023).

The reduced model can be combined with MRI anatomical measurements to derive subject-specific predictions of drug dispersion, following the methodology outlined by Coenen et al. (2019). Sample computations are given for the transient solute evolution associated with the release of a finite dose and with the continuous infusion of a small constant rate. Buoyancy forces alter the mean Lagrangian motion, promoting upward (cranial)/downward (caudal) transport of light/heavy solutes. The comparisons presented in figures 4 and 5 clearly underline the important role of the small drug-to-CSF density differences $10^{-4}≲\left|\rho -\rho_{d}\right|/\rho ≲10^{-2}$, confirming previous clinical observations (Mitchell et al. 1988; Povey et al. 1989; Richardson et al. 1996; Veering et al. 2001; Loubert et al. 2011).

Future refinements of the transport description should account for additional effects, including respiration-induced flow, which is known to prevail in the lumbar region (Aktas et al. 2019; Gutiérrez-Montes et al. 2022), thereby possibly promoting drug dispersion near the injection site. Also important is the effect of the different micro-anatomical features that populate the spinal canal, such as denticulate ligaments, nerve roots and trabeculae (Stockman 2006; Gupta et al. 2008; Pahlavian et al. 2014; Tangen et al. 2015; Haga et al. 2017; Khani et al. 2018; Ayansiji et al. 2023). For instance, the recent experiments of Ayansiji et al. (2023) have shown that the presence of nerve roots significantly promotes tracer dispersion. The effect of trabeculae, which form a continuous weblike structure stretching across the spinal canal (Mortazavi et al. 2018), can be modelled by adding a distributed Brinkman flow resistance term to the momentum equation, as done earlier (Gupta et al. 2008; Tangen et al. 2015; Sincomb et al. 2022). Nerve roots and ligaments, on the other hand, are arranged in quasi-periodic rows aligned along the canal. Their discrete nature may potentially hinder their integration in models based on a slowly varying geometry. Fundamental understanding acquired in connection with oscillatory flows in wavy channels (Guibert, Plouraboué & Bergeon 2010; Alaminos-Quesada et al. 2022, 2023a) and obstacle arrays (House, Lieu & Schwartz 2014; Bhosale, Parthasarathy & Gazzola 2020; Alaminos-Quesada et al. 2023b) can be instrumental to aid these future modelling efforts. In this connection, it is worth mentioning the approximate transport equation proposed recently by Linninger et al. (2023), which incorporates a longitudinal diffusion term with an experimentally fitted diffusivity as a computationally inexpensive means to provide quantification of drug dispersion in the presence of nerve roots.

Additional *in vitro* experiments, similar to those carried out recently (Ayansiji et al. 2023; Moral-Pulido et al. 2023), could be useful in guiding further model refinements. Besides consideration of effects of nerve roots, addressed in the recent work of Ayansiji et al. (2023), these future efforts should specifically consider the quantification of buoyancy-induced flow, with the densities of the working fluids representing the drug and the CSF selected to match the Richardson numbers found in ITDD applications. These experiments will be challenging, because the required density differences are extremely small, so that additional care will be needed to avoid density departures stemming from temperature differences.

Incorporation of pharmacokinetic effects, such as tissue uptake and drug clearance by the blood, which are central to ITDD (Segal & Brunnemann 1989; Sarntinoranont et al. 2003; Kuttler et al. 2010; Linninger et al. 2023), will be necessary to improve the predictive capability of the model in connection with clinical applications. Many drugs have characteristic absorption times of the order of the spinal residence time, so that a non-negligible fraction of the solute deposited in the lumbar region is absorbed along the canal before reaching the cranial cavity. For heavy drugs delivered in an upright position, the case depicted in figures 4(a) and 5(a), the combined effects of buoyancy forces and tissue uptake can be expected to result in drug confinement in the lumbar region, which can be beneficial for analgesic administration. In contrast, buoyancy can promote the dispersion of light drugs towards the cranial cavity, as seen in figures 4(c) and 5(c), thereby limiting uptake rates along the spine and enabling drug delivery to distant intracranial locations.

## Supplementary Material

movie 1

movie 2

movie 3

movie 4

movie 5

movie 6

## Figures and Tables

**Table 1.** A few common intrathecal drugs, with their densities (Nicol & Holdcroft 1992; Lui et al. 1998; Hejtmanek et al. 2011) and associated Richardson numbers $Ri=\left[g\left(\rho -\rho_{d}\right)\right]/\left(\rho \varepsilon^{2}\omega^{2}L\right)$, the latter evaluated with $g=9.81\;m{\;s}^{-2},L=0.6\;m$ and $\rho =1.00059\;g{\;cm}^{-3}$ for two different values of the reduced stroke length ε.

Drug	$\rho d\;(g\;{\text{cm}}^{-3})$	$\frac{\rho -\rho d}{\rho }$	${\mathrm{Ri}}_{\varepsilon =0.04}$	${\mathrm{Ri}}_{\varepsilon =0.02}$
Fentanyl (50 μg ml^−1^)	0.99320	7.386 × 10^−3^	1.963	7.765
Droperidol (2.5 mg ml^−1^)	0.99440	6.186 × 10^−3^	1.601	6.405
Midazolam (1 mg ml^−1^)	0.99970	0.889 × 10^−3^	0.230	0.921
Lidocaine (20 mg ml^−1^)	0.99990	0.690 × 10^−3^	0.178	0.714
Epinephrine (1 mg ml^−1^)	1.00050	0.090 × 10^−3^	0.0236	0.093
Bupivacaine (10 mg ml^−1^)	1.00072	−0.130 × 10^−3^	−0.033	−0.135
Lidocaine CO_2_ (20 mg ml^−1^)	1.00100	−0.410 × 10^−3^	−0.106	−0.424
Morphine (10 mg ml^−1^)	1.00157	−0.979 × 10^−3^	−0.254	−1.014
Meperidine (100 mg ml^−1^)	1.00830	−7.206 × 10^−3^	−1.994	−7.98

## Appendix A. Buoyancy-free velocity description

The solution for the velocity field in the spinal canal in the absence of buoyancy forces was given in our previous publications (Sánchez et al. 2018; Lawrence et al. 2019). A summary of the relevant formulae, needed to quantify the steady-streaming and Stokes-drift velocities appearing in the convective terms in (4.14), is given in this appendix.

The solution to the leading-order problem (4.1)–(4.4) is given by the harmonic functions (4.5), which are repeated here for convenience:

(A1)
u0=ReieitU,v0=ReieitV,w0=ReieitW,p0′=ReeitP′,pˆ0=ReeitPˆ,h0′=ReeitH′.

The complex functions describing the spatial variations of the velocity components can be written as

(A2)
$$U=\frac{dP^{\prime }}{dx}G,$$

(A3)
$$W=\frac{1}{\ell }\frac{\partial \overset{ˆ}{P}}{\partial s}G,$$

(A4)
$$V=-\frac{1}{\ell }\frac{\partial }{\partial x}\left(\ell \frac{dP^{\prime }}{dx}\overset{‾}{h}\int_{0}^{\eta }G\;d\eta \right)-\frac{1}{\ell }\frac{\partial }{\partial s}\left(\frac{1}{\ell }\frac{\partial \overset{ˆ}{P}}{\partial s}\overset{‾}{h}\int_{0}^{\eta }G\;d\eta \right)+\left[\frac{\partial \overset{‾}{h}}{\partial x}\frac{dP^{\prime }}{dx}+\frac{1}{\ell^{2}}\frac{\partial \overset{‾}{h}}{\partial s}\frac{\partial \overset{ˆ}{P}}{\partial s}\right]\eta G,$$

in terms of the auxiliary functions

(A5a,b)
$$G=1-\frac{cos\;h[\Lambda (2\eta \;-\;1)]}{cos\;h\;\Lambda }\;\;\text{and}\;\;\int_{0}^{\eta }G\;d\tilde{\eta }=\eta -\frac{\sinh\left[\Lambda \left(2\eta \;-\;1\right)\right]+\;sinh\;\Lambda }{2\Lambda \;cos\;h\;\Lambda },$$

where

(A6)
$$\Lambda \left(x,s\right)=\frac{\alpha \overset{‾}{h}}{2}\frac{1\;+\;i}{\sqrt{2}}.$$

As in the main text, tildes are used throughout the appendix to denote dummy integration variables. The axial pressure variation is obtained from the boundary-value problem

(A7a,b)
$$\frac{1}{\ell }\frac{d}{dx}\left[\ell \left(\int_{0}^{1}q\;ds\right)\frac{dP^{\prime }}{dx}\right]+\left(k^{2}P^{\prime }+1\right)\int_{0}^{1}\gamma \;ds=0,\;\left\{\begin{array}{ll} P^{\prime }=0 & \;\text{at}\;x=0,\\ \frac{dP^{\prime }}{dx}=0 & \;\text{at}\;x=1, \end{array}\right.$$

involving the volume flux function $\int_{0}^{1}q\;ds$, with

(A8)
$$q\left(x,\;s\right)=\overset{‾}{h}\int_{0}^{1}G\;d\eta =\overset{‾}{h}\left(1-\frac{\tanh\Lambda }{\Lambda }\right).$$

The function $P^{\prime }(x)$ can be used in

(A9)
$$H^{\prime }=\gamma \left(1+k^{2}P^{\prime }\right)$$

to evaluate the canal deformation, and in

(A10)
$$\frac{1}{\ell }\frac{\partial \overset{ˆ}{P}}{\partial s}=-\frac{1}{q}\left[\frac{\partial }{\partial x}\left(\ell \int_{0}^{s}q\;d\tilde{s}\frac{dP^{\prime }}{dx}\right)+\ell \left(k^{2}P^{\prime }+1\right)\int_{0}^{s}\gamma \;ds\right]$$

to evaluate the azimuthal pressure gradient, thereby completing the solution at leading order.

The steady-streaming velocity components ( $u_{SS},v_{SS},\;w_{SS}$ ) are obtained by integration of (4.6)–(4.8) with Ri=0. The functions

(A11)
$$ℱ=-\frac{1}{\ell }\frac{\partial }{\partial x}\left(\ell \left\langle h_{0}^{\prime }u_{0}\right\rangle \right)+\frac{\partial }{\partial \eta }\left(\eta \left\langle u_{0}\frac{\partial h_{0}^{\prime }}{\partial x}\right\rangle \right)-\frac{1}{\ell }\frac{\partial }{\partial s}\left(\left\langle h_{0}^{\prime }w_{0}\right\rangle \right),$$

(A12)
$${ℱ}_{x}=\frac{1}{\ell }\frac{\partial }{\partial x}\left(\ell \left\langle u_{0}^{2}\right\rangle \right)+\frac{1}{\overset{‾}{h}}\frac{\partial }{\partial \eta }\left\langle u_{0}v_{0}\right\rangle +\frac{1}{\ell }\frac{\partial }{\partial s}\left\langle u_{0}w_{0}\right\rangle \;\;-\frac{\eta }{\overset{‾}{h}}\frac{\partial }{\partial \eta }\left\langle \frac{\partial h_{0}^{\prime }}{\partial t}u_{0}\right\rangle -\frac{\partial \overset{‾}{h}}{\partial x}\frac{\eta }{\overset{‾}{h}}\frac{\partial }{\partial \eta }\left\langle u_{0}^{2}\right\rangle -\frac{1}{\ell }\frac{\partial \overset{‾}{h}}{\partial s}\frac{\eta }{\overset{‾}{h}}\frac{\partial }{\partial \eta }\left\langle u_{0}w_{0}\right\rangle +\frac{2}{{\overset{‾}{h}}^{3}\alpha^{2}}\frac{\partial^{2}}{\partial \eta^{2}}\left\langle h_{0}^{\prime }u_{0}\right\rangle$$

and

(A13)
$${ℱ}_{s}=\frac{\partial }{\partial x}\left\langle u_{0}w_{0}\right\rangle +2\frac{\left\langle u_{0}w_{0}\right\rangle }{\ell }\frac{\partial \ell }{\partial x}+\frac{1}{\overset{‾}{h}}\frac{\partial }{\partial \eta }\left\langle v_{0}w_{0}\right\rangle +\frac{1}{\ell }\frac{\partial }{\partial s}\left\langle w_{0}^{2}\right\rangle -\frac{\eta }{\overset{‾}{h}}\frac{\partial }{\partial \eta }\left\langle \frac{\partial h_{0}^{\prime }}{\partial t}w_{0}\right\rangle \;\;-\frac{\partial \overset{‾}{h}}{\partial x}\frac{\eta }{\overset{‾}{h}}\frac{\partial }{\partial \eta }\left\langle u_{0}w_{0}\right\rangle -\frac{1}{\ell }\frac{\partial \overset{‾}{h}}{\partial s}\frac{\eta }{\overset{‾}{h}}\frac{\partial }{\partial \eta }\left\langle w_{0}^{2}\right\rangle +\frac{2}{{\overset{‾}{h}}^{3}\alpha^{2}}\frac{\partial^{2}}{\partial \eta^{2}}\left\langle h_{0}^{\prime }w_{0}\right\rangle$$

appearing on the left-hand sides of (4.6)–(4.8) involve time averages of products of the leading-order functions (A1) that can be evaluated with use of the identity $\left\langle Re\left(e^{i\tau }f_{1}\right)Re\left(e^{i\tau }f_{2}\right)\right\rangle =Re(f_{1}f_{2}^{*})/2$, which applies to any pair of time-independent complex functions $f_{1}$ and $f_{2}$, with the asterisk * denoting complex conjugate. The solution for the steady-streaming velocity can be expressed in the form

(A14)
$$\frac{u_{SS}}{{\overset{‾}{h}}^{2}\alpha^{2}}=\;-\frac{dp_{SS}^{\prime }}{dx}\frac{\left(1\;-\;\eta \right)\eta }{2}+\eta \int_{0}^{\eta }{ℱ}_{x}\;d\tilde{\eta }-\int_{0}^{\eta }{ℱ}_{x}\tilde{\eta }d\tilde{\eta }-\eta \int_{0}^{1}{ℱ}_{x}\left(1-\eta \right)d\eta ,$$

(A15)
$$\frac{w_{SS}}{{\overset{‾}{h}}^{2}\alpha^{2}}=-\frac{1}{\ell }\frac{\partial {\overset{ˆ}{p}}_{SS}}{\partial s}\frac{\left(1\;-\;\eta \right)\eta }{2}+\eta \int_{0}^{\eta }{ℱ}_{S}\;d\tilde{\eta }-\int_{0}^{\eta }{ℱ}_{s}\tilde{\eta }d\tilde{\eta }-\eta \int_{0}^{1}{ℱ}_{S}\left(1-\eta \right)d\eta ,$$

(A16)
$$v_{SS}=\;-\frac{1}{\ell }\frac{\partial }{\partial x}\left(\ell \overset{‾}{h}\int_{0}^{\eta }u_{SS}\;d\tilde{\eta }\right)-\frac{1}{\ell }\frac{\partial }{\partial s}\left(\overset{‾}{h}\int_{0}^{\eta }w_{SS}\;d\tilde{\eta }\right)+\eta \left[\frac{\partial \overset{‾}{h}}{\partial x}u_{SS}+\frac{1}{\ell }\frac{\partial \overset{‾}{h}}{\partial s}w_{SS}\right]\;\;+\eta \left\langle u_{0}\frac{\partial h_{0}^{\prime }}{\partial x}\right\rangle -\frac{1}{\ell }\int_{0}^{\eta }\left[\frac{\partial }{\partial x}\left(\ell \left\langle h_{0}^{\prime }u_{0}\right\rangle \right)+\frac{\partial }{\partial s}\left\langle h_{0}^{\prime }w_{0}\right\rangle \right]d\tilde{\eta }$$

in terms of the axial and azimuthal pressure gradients

(A17)
$$\frac{dp_{SS}^{\prime }}{dx}=\frac{12}{\int_{0}^{1}{\overset{‾}{h}}^{3}\;ds}\int_{0}^{1}\left(\frac{1}{\alpha^{2}}\int_{0}^{1}\left\langle h_{0}^{\prime }u_{0}\right\rangle d\eta -\frac{{\overset{‾}{h}}^{3}}{2}\int_{0}^{1}{ℱ}_{x}\eta (1-\eta )d\eta \right)ds,$$

(A18)
$$\frac{1}{\ell }\frac{\partial {\overset{ˆ}{p}}_{SS}}{\partial s}=\frac{12}{{\overset{‾}{h}}^{3}}\frac{\partial }{\partial x}\left[\ell \int_{0}^{s}\left(\frac{1}{\alpha^{2}}\int_{0}^{1}\left\langle h_{0}^{\prime }u_{0}\right\rangle d\eta -\frac{{\overset{‾}{h}}^{3}}{2}\int_{0}^{1}{ℱ}_{x}\eta (1-\eta )d\eta -\frac{{\overset{‾}{h}}^{3}}{12}\frac{dp_{SS}^{\prime }}{dx}\right)d\tilde{s}\right]\;\;+\frac{12}{{\overset{‾}{h}}^{3}}\left(\frac{1}{\alpha^{2}}\int_{0}^{1}\left\langle h_{0}^{\prime }w_{0}\right\rangle d\eta -\frac{{\overset{‾}{h}}^{3}}{2}\int_{0}^{1}{ℱ}_{s}\eta (1-\eta )d\eta \right),$$

which complete the determination of the steady-streaming velocity. On the other hand, the Stokes-drift velocity components, which provide an additional contribution to the time-averaged Lagrangian drift driving convective transport in the slow time scale, can be expressed in the form

(A19)
$$u_{SD}=\frac{1}{\overset{‾}{h}}\left\{\langle u_{0}h_{0}^{\prime }\rangle +\frac{1}{\ell }\frac{\partial }{\partial s}\left(\overset{‾}{h}\left\langle u_{0}\int w_{0}\;dt\right\rangle \right)\right\}\;\;+\frac{1}{\overset{‾}{h}}\frac{\partial }{\partial \eta }\langle u_{0}\left[\int v_{0}\;dt-\eta \left(h_{0}^{\prime }+\frac{1}{\ell }\frac{\partial \overset{‾}{h}}{\partial s}\int w_{0}\;dt\right)\right]\rangle ,$$

(A20)
$$v_{SD}=\frac{1}{\ell }\frac{\partial }{\partial x}\left(\ell \langle v_{0}\int u_{0}\;dt\rangle \right)+\frac{1}{\ell }\frac{\partial }{\partial s}\langle v_{0}\int w_{0}\;dt\rangle \;\;-\frac{\eta }{\overset{‾}{h}}\frac{\partial }{\partial \eta }\langle v_{0}\left(h_{0}^{\prime }+\frac{\partial \overset{‾}{h}}{\partial x}\int u_{0}\;dt+\frac{1}{\ell }\frac{\partial \overset{‾}{h}}{\partial s}\int w_{0}\;dt\right)\rangle ,$$

(A21)
$$w_{SD}=\frac{1}{\overset{‾}{h}}\left[\left\langle w_{0}h_{0}^{\prime }\right\rangle +\frac{\partial }{\partial x}\left(\overset{‾}{h}\langle w_{0}\int u_{0}\;dt\rangle \right)\right]\;\;+\frac{1}{\overset{‾}{h}}\frac{\partial }{\partial \eta }\left\langle w_{0}\left[\int v_{0}\;dt-\eta \left(h_{0}^{\prime }+\frac{\partial \overset{‾}{h}}{\partial x}\int u_{0}\;dt\right)\right]\right\rangle ,$$

where the different time averages can be evaluated with use of (A1) and associated antiderivatives $\int u_{0}\;dt=Re\left(e^{it}U\right),\;\int v_{0}\;dt=Re\left(e^{it}V\right)$, and $\int w_{0}\;dt=Re\left(e^{it}W\right)$.
